# Sylvatic dengue virus type 4 in *Aedes aegypti* and *Aedes albopictus* mosquitoes in an urban setting in Peninsular Malaysia

**DOI:** 10.1371/journal.pntd.0007889

**Published:** 2019-11-15

**Authors:** Nur Alia Johari, Kenny Voon, Shen Yung Toh, Lokman Hakim Sulaiman, Ivan Kok Seng Yap, Patricia Kim Chooi Lim

**Affiliations:** 1 Institute for Research, Development and Innovation (IRDI), International Medical University, Kuala Lumpur, Malaysia; 2 Pathology Division, School of Medicine, International Medical University, Kuala Lumpur, Malaysia; 3 Department of Community Medicine, School of Medicine, International Medical University, Kuala Lumpur, Malaysia; 4 Sarawak Research and Development Council, Ministry of Education, Science and Technological Research, Sarawak, Malaysia; University of Texas Medical Branch, UNITED STATES

## Abstract

Dengue fever is endemic in Malaysia, contributing to significant economic and health burden in the country. *Aedes aegypti* and *Ae*. *albopictus* are the main vectors of the dengue virus (DENV), which circulates in sylvatic and human transmission cycles and has been present in Malaysia for decades. The study investigated the presence and distribution of DENV in urban localities in the Klang Valley, Peninsular Malaysia. A total of 364 *Ae*. *aegypti* and 1,025 *Ae*. *albopictus* larvae, and 10 *Ae*. *aegypti* and 42 *Ae*. *albopictus* adult mosquitoes were screened for the presence of DENV. In total, 31 (2.2%) samples were positive, of which 2 *Ae*. *albopictus* larvae were co-infected with two serotypes, one with DENV-2 and DENV-3 and the other with DENV-3 and DENV-4. Phylogenetic analysis determined that the isolates belonged to DENV-1 genotype I (1 *Ae*. *aegypti* adult), DENV-2 (1 *Ae*. *albopictus* larva), DENV-3 genotype V (3 *Ae*. *aegypti* larvae and 10 *Ae*. *albopictus* larvae) and DENV-4 genotype IV (6 *Ae*. *aegypti* larvae and 12 *Ae*. *albopictus* larvae), a sylvatic strain of DENV-4 which was most closely related with sylvatic strains isolated from arboreal mosquitoes and sentinel monkeys in Peninsular Malaysia in the 1970s. All four DENV serotypes were co-circulating throughout the study period. The detection of a sylvatic strain of DENV-4 in *Ae*. *aegypti* and *Ae*. *albopictus* mosquitoes in urban areas in Peninsular Malaysia highlights the susceptibility of these vectors to infection with sylvatic DENV. The infectivity and vector competence of these urban mosquitoes to this strain of the virus needs further investigation, as well as the possibility of the emergence of sylvatic virus into the human transmission cycle.

## Author summary

Dengue is a mosquito-borne disease that has re-emerged as a major public health problem in the 21^st^ Century. The four serotypes of dengue virus are capable of infecting humans with a range of symptoms and clinical outcomes, with the change in predominating serotypes often responsible for new dengue outbreaks. In this study, we surveyed urban sites in the Klang Valley, Malaysia, for *Aedes aegypti* and *Aedes albopictus* mosquito vectors and confirmed the presence of all four serotypes of the virus. We detected the presence of genotype V of dengue serotype 3, which has previously not been reported in the country, as well as a sylvatic strain of dengue serotype 4 that was closely related to strains obtained from forest-dwelling mosquito vectors and sentinel monkeys in Malaysia. The detection of a sylvatic strain of dengue virus in urban vectors raises questions over the risk of virus transmission to humans, especially with recent reports of severe clinical cases as a result of infection with sylvatic dengue virus serotypes. Further studies will be needed to determine the source and origins of these strains as well as the severity of dengue disease upon infection, especially with the development of new dengue vaccines.

## Introduction

Dengue is a mosquito-borne viral infection that has re-emerged as a significant public health problem worldwide, especially in tropical and subtropical regions within which the disease is endemic [1]. Every year, approximately 96 million cases are reported worldwide, with the Asian region accounting for 70% of all cases [2]. Despite efforts to contain its spread, the incidence of dengue infections has increased 30-fold over the last 50 years, moving across borders into regions that had previously had little to no exposure to the disease [3]. Malaysia is no exception, and has experienced an exponential rise in dengue cases from less than 20,000 in 2011 to a historic high of 120,836 cases in 2015 [4]. Dengue virus (DENV) is transmitted to humans primarily by infected *Aedes aegypti* and *Ae*. *albopictus* mosquitoes that are widely distributed in tropical regions, especially in Southeast Asia, and are also the main vectors of chikungunya (CHIKV) [5] and Zika virus (ZIKV) [6].

DENV is a *Flavivirus* with four antigenically distinct serotypes, namely DENV serotype 1 to serotype 4 (DENV-1 to DENV-4) [7]. The virus circulates in two distinct transmission cycles; the sylvatic cycle between the vector mosquito and non-human primates, and the human transmission cycle involving domestic and peridomestic *Aedes* mosquitoes [8]. Sylvatic strains of DENV serotypes 1, 2 and 4 have been isolated from sentinel monkeys in Peninsular Malaysia, with the presence of sylvatic DENV-3 inferred based on the seroconversion of sentinel monkeys [9,10]. A sylvatic strain of DENV-4 was also isolated from canopy-dwelling *Ae*. *niveus* mosquitoes in Malaysia in 1975 [11]. In Africa, only sylvatic DENV-2 has been isolated thus far with the strains found to be phylogenetically distinct from other circulating DENV-2 strains [12]. Compared to sylvatic DENV strains detected in Africa, phylogenetic studies have noted the presence of a wider diversity of sylvatic DENV strains in Malaysia, suggesting that a DENV ancestor most likely originated in the Asian region and subsequently diverged into at least four DENV serotypes that are currently circulating [13].

In 2008, a patient who developed dengue haemorrhagic fever (DHF) in Peninsular Malaysia was found to be infected with a sylvatic DENV-2 strain [14]. A sylvatic DENV-1 virus was also isolated from a patient with dengue fever in the country, and it was suggested that the infection could be due to a rare spillover event as a result of contact with infected forest-dwelling vectors [15]. These findings indicate that in this region, sylvatic DENV is being maintained in a zoonotic cycle, as it has been for the last four decades [16]. Furthermore, a sylvatic DENV-2 strain was isolated in West Africa from a man with DHF. This isolate was however found to be from a completely different lineage of the virus detected in Malaysia [17]. Thus far, the sylvatic strain of DENV-4 has yet to be reported in humans, with only three known sequences that were successfully isolated in Malaysia [18]. These reports suggest that sylvatic strains of DENV are capable of causing clinical disease in humans and, with the appropriate conditions, may emerge into an urban transmission cycle without requiring significant adaptation to the human host. There exist considerable genetic differences between human and sylvatic DENV strains. However, studies by Vasilakis and colleagues comparing human and sylvatic strains of DENV-2 [19,20] and DENV-4 [21] found no significant difference in the replication kinetics of both strains of each serotype, further highlighting the high possibility of re-emergence of sylvatic strains into the human transmission cycle. Despite the growing impact of dengue infections amongst human populations, there remains a lack of information on the presence and distribution of DENV strains in *Aedes* mosquitoes in Malaysia. In this study we aimed to investigate the presence and distribution of DENV in urban localities in the Klang Valley, Peninsular Malaysia. We report the presence of all four DENV serotypes, including a sylvatic strain of DENV-4, in field captured *Ae*. *aegypti* and *Ae*. *albopictus* mosquitoes.

## Methods

### Ethics statement

This study has obtained ethical approval from the Medical Research & Ethics Committee, Ministry of Health Malaysia (Reference number: NMRR-15-2128-28012).

### Sampling

**Fig 1:**
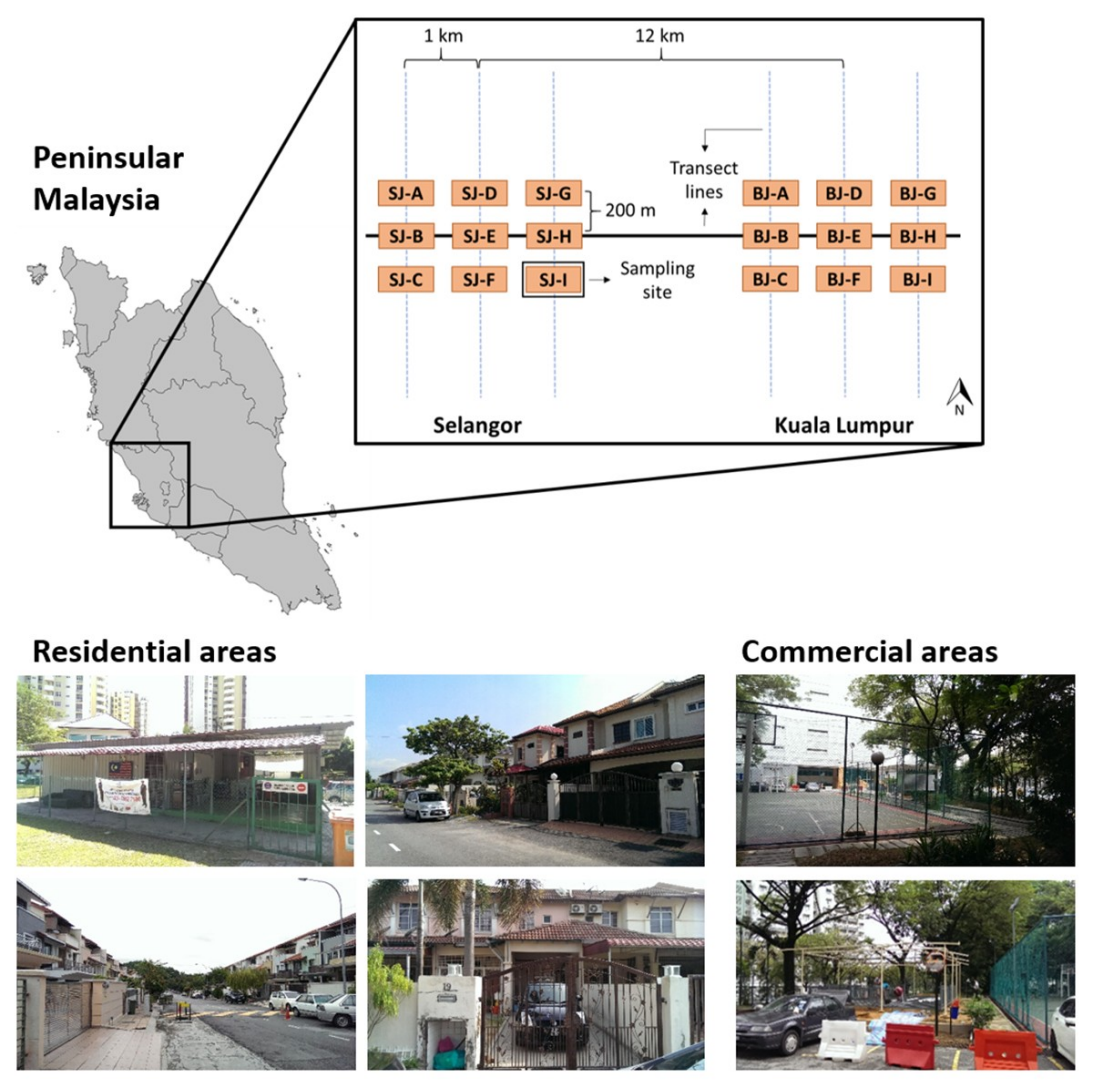
Map of Peninsular Malaysia with a transect line diagram of the sampling sites selected within Selangor and Kuala Lumpur.

From May 2016 to April 2017, *Aedes* larvae and adult mosquitoes were collected from two urban areas (Selangor and Kuala Lumpur) in the Klang Valley, Malaysia. Localities within Selangor and Kuala Lumpur were selected along a transect line extending from East to West. In total, nine sites were selected for sampling in each area (labelled A to I) for a total of 18 sampling sites. The sampling regimen used included one central site per locality (SJ-E and BJ-E) set approximately 12 km apart. Each area was segmented into three parallel lines 1 km apart from North to South. A series of three sampling sites, each at least 200 m apart, were identified at each segmented line (Fig 1). Table 1 shows the coordinates of each site and their associated land cover. Prior permission was obtained from owners and residents for the study, which was conducted on private land and residences. Samples were collected at monthly intervals using ovitraps for larvae and light traps for adult mosquitoes. Every month, 30 ovitraps were set in shaded locations indoors and outdoors and collected after five days. One light trap was set at each site and collected after 24 hours. In total, 9 light traps and 270 ovitraps were collected from each area every month and transported back to the laboratory for processing. A trap was recorded as positive when it contained *Aedes* mosquitoes of any species. Each sample was identified by species using keys by Rueda [22] and coded by month, trap and site number, and subsequently stored in -80°C prior to further processing [23].

**Table 1:** Land cover and GPS coordinates of sampling sites within Selangor and Kuala Lumpur.

Area	Site	Land cover	GPS coordinates
**Selangor**	SJ-A	Residential area (individual house)	3° 2'56.59"N, 101°34'54.63"E
	SJ-B	Residential area (individual house)	3° 2'52.58"N, 101°34'57.80"E
	SJ-C	Residential area (individual house)	3° 2'54.06"N, 101°34'46.64"E
	SJ-D	Residential area (individual house)	3° 3'20.19"N, 101°35'11.06"E
	SJ-E	Residential area (individual house)	3° 3'10.01"N, 101°35'8.95"E
	SJ-F	Residential area (park)	3° 3'10.52"N, 101°35'14.67"E
	SJ-G	Residential area (individual house)	3° 3'25.48"N, 101°36'7.92"E
	SJ-H	Residential area (individual house)	3° 3'19.58"N, 101°35'57.87"E
	SJ-I	Residential area (individual house)	3° 3'7.66"N, 101°36'14.72"E
**Kuala Lumpur**	BJ-A	Residential area (individual house)	3° 4'7.79"N, 101°40'56.36"E
	BJ-B	Residential area (individual house)	3° 3'36.80"N, 101°40'31.83"E
	BJ-C	Residential area (individual house)	3° 3'19.68"N, 101°40'34.27"E
	BJ-D	Commercial area	3° 3'37.14"N, 101°41'11.97"E
	BJ-E	Commercial area	3° 3'26.89"N, 101°41'17.18"E
	BJ-F	Residential area (apartment)	3° 3'7.76"N, 101°41'13.85"E
	BJ-G	Residential area (individual house)	3° 4'19.56"N, 101°41'17.05"E
	BJ-H	Residential area (individual house)	3° 4'15.64"N, 101°41'54.57"E
	BJ-I	Residential area (individual house)	3° 3'58.13"N, 101°41'59.31"E

### Detection of DENV in larvae and adult mosquitoes

cDNA synthesis was performed as previously described [24]. For detection of DENV, nested PCR was carried out as previously described [25–27]. In brief, a total of 1,441 field-caught *Aedes* larvae and adult mosquitoes were individually harvested for RNA and subjected to nested PCR. The first and second round of PCR amplification was carried out using the DENV consensus primers Dcon [25], D1 and D2 [26]. To determine the DENV serotype present, the amplified DNA sequence was subjected to multiplex PCR using the same forward D1 primer and four reverse primers TS1, TS2, TS3 and TS4 for each serotype DENV-1, DENV-2, DENV-3 and DENV-4, respectively [26]. The primers used for PCR amplification are detailed in S1 Table. Positive controls for each DENV serotype (DENV-1-DENV-4) as detailed in S2 Table and a negative control (nuclease-free water) were included separately. All PCR products were separated by agarose gel electrophoresis, followed by sequencing the positive PCR products. The expected sizes of the amplified products were 429nt for DENV-1, 70nt for DENV-2, 240nt for DENV-3 and 351nt for DENV-4.

### Phylogenetic analysis

Phylogenetic analysis was conducted in MEGA X [28] using the Maximum Likelihood (ML) method based on the Tamura-Nei model [29] with 1,000 bootstrap resampling. Each sequence was analysed based on the nucleotide sequences of the capsid pre-membrane (CprM) gene to determine the genotype and genetic relationship of each strain. Reference sequences of DENV genotypes from different years and geographical regions were included in order to characterise the genotypes of each sequence. Sylvatic strains available for each serotype were also included in the analysis.

## Results

### Sample collection

In total, 15,994 *Aedes* larvae were collected using ovitraps, of which 837 (5.2%) were *Ae*. *aegypti* and 15,157 (94.8%) were *Ae*. *albopictus*. For the adult mosquito samples, the majority were *Ae*. *albopictus*, comprising 75 (76.5%) of the 98 field collected samples. Due to the large number of *Ae*. *albopictus* larvae collected, one sample was randomly selected from every positive ovitrap at bimonthly intervals for a total 1,025 *Ae*. *albopictus* larvae screened. All adult mosquitoes (N = 52) and *Ae*. *aegypti* larvae (N = 364) collected from the same bimonthly intervals were screened.

### DENV serotype detection

Overall, DENV was detected in 30 (2.2%) samples out of the 1,389 *Aedes* larvae (Tables 2 and 3) tested. Out of the 52 adult *Aedes* mosquitoes screened, only one (1.9%) out of 52 samples was positive for DENV (Tables 4 and 5). All four DENV serotypes were detected during the study period, where the most common serotype was DENV-4 (n = 18), followed by DENV-3 (n = 13) and one sample each that were RNA-positive for DENV-1 and DENV-2. The DENV-1 sequence (USJT8G/2017) was detected in an adult *Ae*. *aegypti*, the sole positive sample (1.92%) out of the 52 adult mosquitoes screened for DENV. The DENV-2 sequence (BJI29-2/2016) was detected in an *Ae*. *albopictus* larva from Kuala Lumpur, which was also co-infected with DENV-4 (BJI29-4/2016). Another *Ae*. *albopictus* larva from the same area was also positive for both DENV-3 (BJD2-3/2016) and DENV-4 (BJD2-4/2016). Out of the 18 larvae positive for DENV-4, 3 *Ae*. *aegypti* larvae in December 2016 as well as another 3 in April 2016 were collected from the same ovitrap set within site SJ-A. The rest of the 24 positive larvae were collected from different individual traps over the field collection period. The details of each sequence and their corresponding GenBank accession numbers (MH377083-115) are shown in S3 Table.

**Table 2:** DENV detected in *Aedes* larvae collected in Selangor from June 2016 to April 2017.

Year	2016				2017		Total
Month	June	Aug	Oct	Dec	Feb	April	
**SJ-A**							
***Ae*. *aegypti***							
No. collected	0	0	0	54	5	21	80
No. positive	0	0	0	2 (DENV-3); 3 (DENV-4)	0	1 (DENV-3); 3 (DENV-4)	9
***Ae*. *albopictus***							
No. collected	37	0	0	29	0	12	78
No. positive	0	0	0	0	0	0	0
**SJ-B**							
***Ae*. *aegypti***							
No. collected	7	0	0	8	0	4	19
No. positive	0	0	0	0	0	0	0
***Ae*. *albopictus***							
No. collected	33	7	36	184	37	5	302
No. positive	0	0	1 (DENV-3)	0	0	0	1
**SJ-C**							
***Ae*. *aegypti***							
No. collected	0	0	33	0	1	0	34
No. positive	0	0	0	0	0	0	0
***Ae*. *albopictus***							
No. collected	2	0	11	53	20	23	109
No. positive	0	0	0	0	0	0	0
**SJ-D**							
***Ae*. *aegypti***							
No. collected	0	0	0	0	0	0	0
No. positive	0	0	0	0	0	0	0
***Ae*. *albopictus***							
No. collected	48	1	7	41	1	11	109
No. positive	0	0	0	0	0	0	0
**SJ-E**							
***Ae*. *aegypti***							
No. collected	0	0	0	0	0	0	0
No. positive	0	0	0	0	0	0	0
***Ae*. *albopictus***							
No. collected	4	27	13	10	0	0	54
No. positive	0	0	0	0	0	0	0
**SJ-F**							
***Ae*. *aegypti***							
No. collected	0	0	0	0	1	1	2
No. positive	0	0	0	0	0	0	0
***Ae*. *albopictus***							
No. collected	205	236	25	238	205	207	1,116
No. positive	1 (DENV-4)	0	0	0	0	0	1
**SJ-G**							
***Ae*. *aegypti***							
No. collected	0	0	1	1	0	1	3
No. positive	0	0	0	0	0	0	0
***Ae*. *albopictus***							
No. collected	26	51	24	155	37	144	437
No. positive	0	0	1 (DENV-4)	0	0	0	1
**SJ-H**							
***Ae*. *aegypti***							
No. collected	2	0	16	3	0	4	25
No. positive	0	0	0	0	0	0	0
***Ae*. *albopictus***							
No. collected	51	2	1	92	35	9	190
No. positive	0	0	0	0	0	0	0
**SJ-I**							
***Ae*. *aegypti***							
No. collected	0	0	0	0	0	0	0
No. positive	0	0	0	0	0	0	0
***Ae*. *albopictus***							
No. collected	49	44	27	133	129	128	510
No. positive	0	2 (DENV-3)	0	0	0	0	2

**Table 3:** DENV detected in *Aedes* larvae collected in Kuala Lumpur from June 2016 to April 2017.

Year	2016				2017		Total
Month	June	Aug	Oct	Dec	Feb	April	
**BJ-A**							
***Ae*. *aegypti***							
No. collected	0	2	0	0	9	3	14
No. positive	0	0	0	0	0	0	0
***Ae*. *albopictus***							
No. collected	149	32	10	74	55	197	517
No. positive	1 (DENV-4)	0	0	1 (DENV-4)	0	0	2
**BJ-B**							
***Ae*. *aegypti***							
No. collected	0	0	0	0	0	1	1
No. positive	0	0	0	0	0	0	0
***Ae*. *albopictus***							
No. collected	22	1	2	16	0	7	48
No. positive	1 (DENV-3)	0	0	0	0	0	1
**BJ-C**							
***Ae*. *aegypti***							
No. collected	0	0	0	0	0	0	0
No. positive	0	0	0	0	0	0	0
***Ae*. *albopictus***							
No. collected	116	68	28	79	9	91	391
No. positive	0	0	0	0	0	0	0
**BJ-D**							
***Ae*. *aegypti***							
No. collected	0	0	8	0	0	0	8
No. positive	0	0	0	0	0	0	0
***Ae*. *albopictus***							
No. collected	232	75	258	95	254	225	1,139
No. positive	1 (DENV-3); 1 (DENV-4)	0	0	0	0	0	2
**BJ-E**							
***Ae*. *aegypti***							
No. collected	0	0	0	2	0	14	16
No. positive	0	0	0	0	0	0	0
***Ae*. *albopictus***							
No. collected	166	299	237	317	281	221	1,521
No. positive	0	1 (DENV-4)	0	0	0	0	1
**BJ-F**							
***Ae*. *aegypti***							
No. collected	0	0	0	29	5	5	39
No. positive	0	0	0	0	0	0	0
***Ae*. *albopictus***							
No. collected	19	0	0	12	0	1	32
No. positive	1 (DENV-3)	0	0	0	0	0	1
**BJ-G**							
***Ae*. *aegypti***							
No. collected	0	4	3	5	0	2	14
No. positive	0	0	0	0	0	0	0
***Ae*. *albopictus***							
No. collected	60	81	18	101	51	99	410
No. positive	3 (DENV-3); 1 (DENV-4)	1 (DENV-4)	1 (DENV-4)	0	0	0	6
**BJ-H**							
***Ae*. *aegypti***							
No. collected	40	81	0	2	5	1	129
No. positive	0	2 (DENV-4)	0	0	0	0	0
***Ae*. *albopictus***							
No. collected	50	93	1	36	62	102	344
No. positive	1 (DENV-3)	0	0	0	0	0	1
**BJ-I**							
***Ae*. *aegypti***							
No. collected	2	0	48	14	0	6	70
No. positive	0	0	0	0	0	0	0
***Ae*. *albopictus***							
No. collected	52	43	53	167	35	162	512
No. positive	1 (DENV-2); 1 (DENV-4)	0	0	0	0	0	2

**Table 4:** DENV detected in adult *Aedes* mosquitoes collected in Selangor from June 2016 to April 2017.

Year	2016				2017		Total
Month	June	Aug	Oct	Dec	Feb	April	
**SJ-A**							
***Ae*. *aegypti***							
No. collected	0	0	0	0	0	0	0
No. positive	0	0	0	0	0	0	0
***Ae*. *albopictus***							
No. collected	0	0	0	0	0	0	0
No. positive	0	0	0	0	0	0	0
**SJ-B**							
***Ae*. *aegypti***							
No. collected	0	0	0	0	0	0	0
No. positive	0	0	0	0	0	0	0
***Ae*. *albopictus***							
No. collected	0	0	0	0	0	0	0
No. positive	0	0	0	0	0	0	0
**SJ-C**							
***Ae*. *aegypti***							
No. collected	0	0	0	0	0	0	0
No. positive	0	0	0	0	0	0	0
***Ae*. *albopictus***							
No. collected	0	0	0	0	0	1	1
No. positive	0	0	0	0	0	0	0
**SJ-D**							
***Ae*. *aegypti***							
No. collected	0	0	0	0	0	0	0
No. positive	0	0	0	0	0	0	0
***Ae*. *albopictus***							
No. collected	1	0	0	0	0	0	1
No. positive	0	0	0	0	0	0	0
**SJ-E**							
***Ae*. *aegypti***							
No. collected	0	0	0	0	0	0	0
No. positive	0	0	0	0	0	0	0
***Ae*. *albopictus***							
No. collected	0	0	0	0	0	0	0
No. positive	0	0	0	0	0	0	0
**SJ-F**							
***Ae*. *aegypti***							
No. collected	0	0	0	0	0	0	0
No. positive	0	0	0	0	0	0	0
***Ae*. *albopictus***							
No. collected	1	1	0	1	0	2	5
No. positive	0	0	0	0	0	0	0
**SJ-G**							
***Ae*. *aegypti***							
No. collected	0	0	0	0	0	0	0
No. positive	0	0	0	0	0	0	0
***Ae*. *albopictus***							
No. collected	0	0	0	1	0	0	1
No. positive	0	0	0	0	0	0	0
**SJ-H**							
***Ae*. *aegypti***							
No. collected	0	0	0	0	1	0	1
No. positive	0	0	0	0	1 (DENV-1)	0	0
***Ae*. *albopictus***							
No. collected	2	0	0	0	0	0	2
No. positive	0	0	0	0	0	0	0
**SJ-I**							
***Ae*. *aegypti***							
No. collected	0	1	0	0	0	0	1
No. positive	0	0	0	0	0	0	0
***Ae*. *albopictus***							
No. collected	0	0	0	0	1	1	2
No. positive	0	0	0	0	0	0	0

**Table 5:** DENV detected in adult *Aedes* mosquitoes collected in Kuala Lumpur from June 2016 to April 2017.

Year	2016				2017		Total
Month	June	Aug	Oct	Dec	Feb	April	
**BJ-A**							
***Ae*. *aegypti***							
No. collected	0	0	0	0	0	0	0
No. positive	0	0	0	0	0	0	0
***Ae*. *albopictus***							
No. collected	0	0	0	0	0	0	0
No. positive	0	0	0	0	0	0	0
**BJ-B**							
***Ae*. *aegypti***							
No. collected	0	0	0	0	0	0	0
No. positive	0	0	0	0	0	0	0
***Ae*. *albopictus***							
No. collected	1	0	0	0	0	1	2
No. positive	0	0	0	0	0	0	0
**BJ-C**							
***Ae*. *aegypti***							
No. collected	0	0	0	0	0	0	0
No. positive	0	0	0	0	0	0	0
***Ae*. *albopictus***							
No. collected	1	0	0	0	0	0	1
No. positive	0	0	0	0	0	0	0
**BJ-D**							
***Ae*. *aegypti***							
No. collected	0	0	0	1	0	1	2
No. positive	0	0	0	0	0	0	0
***Ae*. *albopictus***							
No. collected	2	0	5	2	4	0	13
No. positive	0	0	0	0	0	0	0
**BJ-E**							
***Ae*. *aegypti***							
No. collected	0	0	0	0	0	1	1
No. positive	0	0	0	0	0	0	0
***Ae*. *albopictus***							
No. collected	4	0	2	3	2	1	12
No. positive	0	0	0	0	0	0	0
**BJ-F**							
***Ae*. *aegypti***							
No. collected	0	0	0	0	0	0	0
No. positive	0	0	0	0	0	0	0
***Ae*. *albopictus***							
No. collected	0	0	0	0	0	0	0
No. positive	0	0	0	0	0	0	0
**BJ-G**							
***Ae*. *aegypti***							
No. collected	0	1	0	0	0	0	1
No. positive	0	0	0	0	0	0	0
***Ae*. *albopictus***							
No. collected	0	0	0	1	0	0	1
No. positive	0	0	0	0	0	0	0
**BJ-H**							
***Ae*. *aegypti***							
No. collected	2	0	0	0	1	0	3
No. positive	0	0	0	0	0	0	0
***Ae*. *albopictus***							
No. collected	0	0	0	0	0	0	0
No. positive	0	0	0	0	0	0	0
**BJ-I**							
***Ae*. *aegypti***							
No. collected	0	0	0	1	0	0	1
No. positive	0	0	0	0	0	0	0
***Ae*. *albopictus***							
No. collected	1	0	0	1	0	0	2
No. positive	0	0	0	0	0	0	0

### Phylogenetic analysis

**Fig 2:**
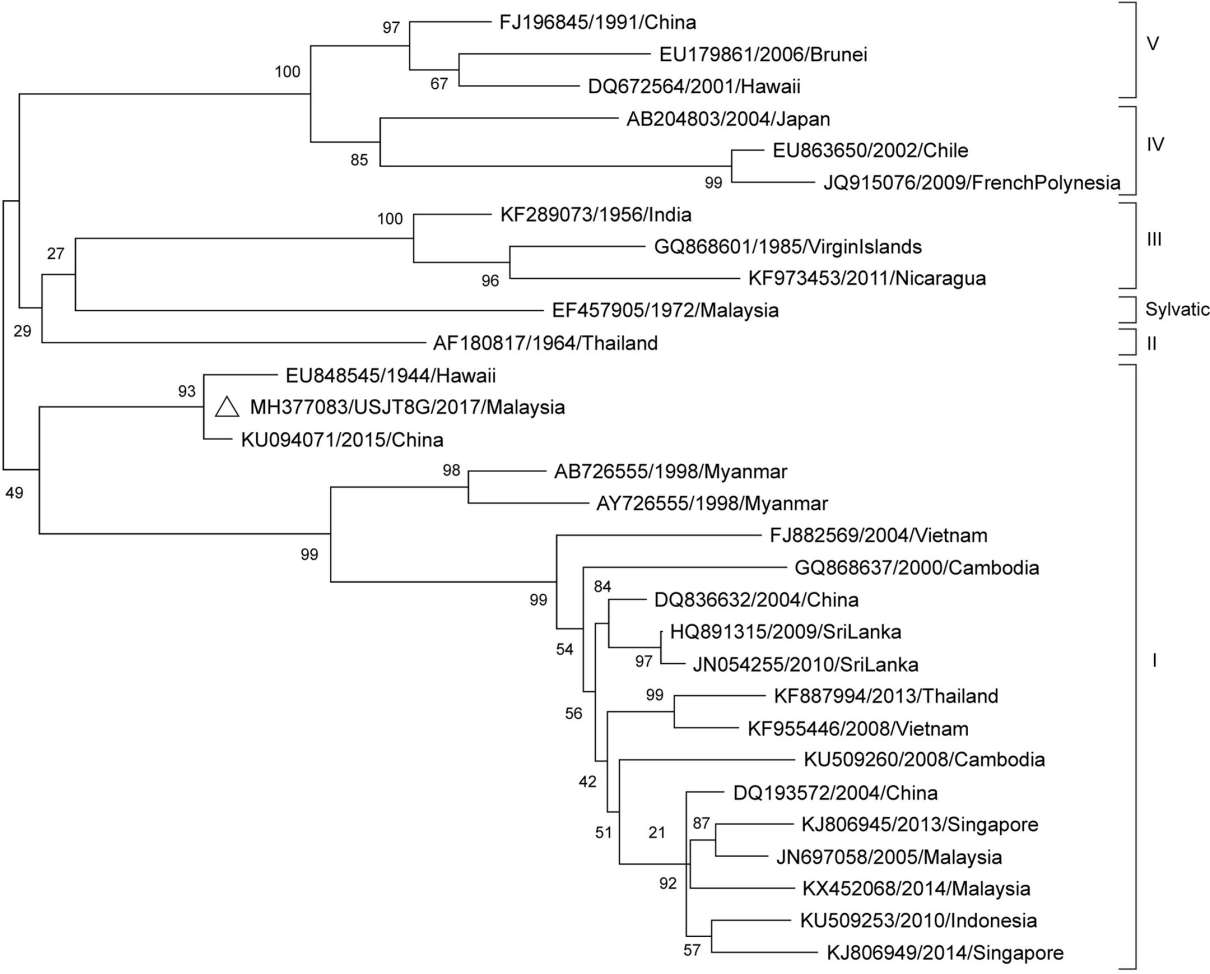
Phylogenetic analysis of the CprM gene sequences of DENV-1 isolates from the Klang Valley. Sample sequence of DENV-1 (429 nt) from adult *Ae*. *aegypti* collected from Selangor in this study (marked with a white triangle) is labelled with the GenBank accession number, sample ID and year of collection. Bootstrap values (1,000 replicates) are shown next to the branches. Taxa of reference sequences are labelled as the GenBank accession numbers, year and country of sample collection.

**Fig 3:**
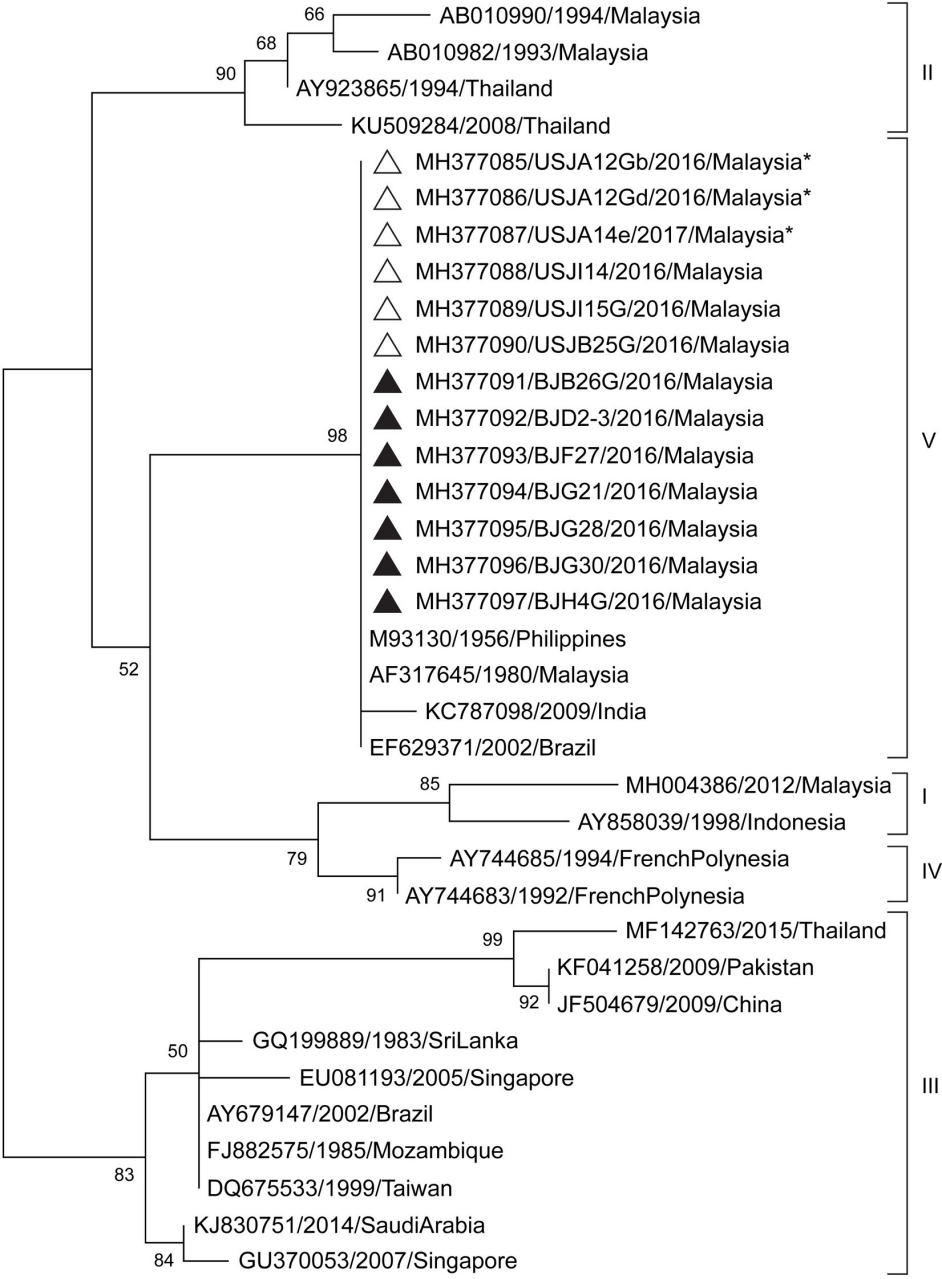
Phylogenetic analysis of the CprM gene sequences of DENV-3 isolates from the Klang Valley. Sample sequences of DENV-3 (240 nt) from *Ae*. *aegypti* (indicated with an asterisk *) and *Ae*. *albopictus* collected from Selangor (indicated with a white triangle) and Kuala Lumpur (indicated with a black triangle) in this study are labelled with the GenBank accession number, sample ID and year of collection. Bootstrap values (1,000 replicates) are shown next to the branches. Taxa of reference sequences are labelled as the GenBank accession numbers, year and country of sample collection.

**Fig 4:**
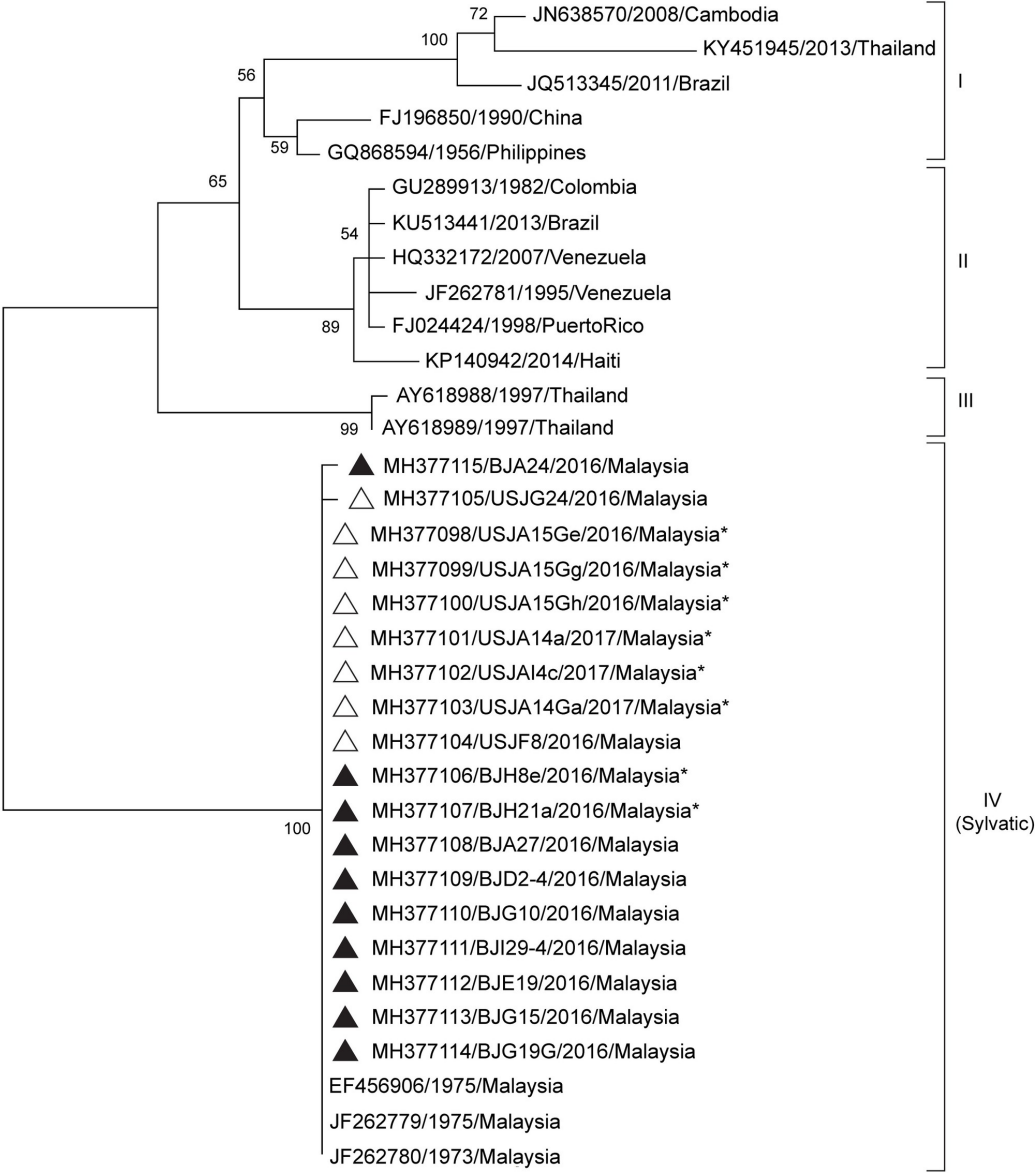
Phylogenetic analysis of the CprM gene sequences of DENV-4 isolates from the Klang Valley. Sample sequences of DENV-4 (351 nt) from *Ae*. *aegypti* (indicated with an asterisk *) and *Ae*. *albopictus* collected from Selangor (indicated with a white triangle) and Kuala Lumpur (indicated with a black triangle) in this study are labelled with the GenBank accession number, sample ID and year of collection. Bootstrap values (1,000 replicates) are shown next to the branches. Taxa of reference sequences are labelled as the GenBank accession numbers, year and country of sample collection.

Phylogenetic trees of the CprM gene sequences for each DENV serotype and reference sequences for the respective genotypes are shown in Figs 2–4. The DENV-1 sequence USJT8G/2017 obtained in this study was analysed with reference sequences from genotypes I to V (Fig 2), as well as one sylvatic strain isolated from a sentinel monkey in Malaysia in 1975 (GenBank accession number: EF457905) [30]. The DENV-1 strain was obtained from an adult *Ae*. *aegypti* mosquito collected in Selangor in 2017. Phylogenetic analysis revealed that this strain belonged to genotype I, one of the most prevalent genotypes in Southeast Asia [31]. The strain clustered closely with two other isolates, namely one from Hawaii in 1944 (EU848545) and another more recent isolate from China in 2015 (KU094071). Many DENV-1 strains isolated between the 1940s and 2010s from Southeast Asia also belong to this genotype, including Malaysian isolates from Johor Bahru (KX452068) and the Klang Valley (JN697058).

The DENV-2 sequence BJI29-2/2016 obtained in this study was analysed with 32 reference sequences from six genotypes of DENV-2, namely the Asian I, Asian II, American/Asian, American and Cosmopolitan genotypes and included sylvatic strains isolated in Malaysia. Based on this analysis, we can confirm that the DENV strain sequenced from an *Ae*. *albopictus* larva collected from Kuala Lumpur in 2016 belonged to serotype 2. However, we are unable to conduct any further analysis to determine the genotype of the sequence due to the short sequence length.

The DENV-3 (n = 13) sequences obtained in this study were analysed with 22 reference sequences (Fig 3) from genotypes I to V. Based on this analysis, all the sample sequences clustered with strains from genotype V, including with the first DENV-3 genotype V strain isolated in the Philippines in 1956 (M93130) [32,33] as well as with isolates that were detected in India in 2009 (KC787098) and in Brazil in 2002 (EF629371). Genotype V is represented by early Asian strains and were recently detected among patients during outbreaks in Brazil and Colombia [32].

The DENV-4 (n = 18) sequences obtained in this study were analysed with 16 reference sequences (Fig 4) from genotypes I to III as well as three sylvatic strains (genotype IV). Genotype III is specifically found in neighbouring Thailand, with genotypes I and II represented by strains from Southeast Asia, Sri Lanka, the Caribbean and the Americas [13]. Phylogenetic analysis revealed that the sequences obtained from both *Ae*. *aegypti* and *Ae*. *albopictus* in this study belonged to the sylvatic genotype, clustering with early sylvatic isolates collected in the 1970s from the forest-dwelling *Ae*. *niveus* (EF457906), and from sentinel monkeys (JF262780 and JF262779) in Peninsular Malaysia [9].

## Discussion

This study reports the detection of four DENV serotypes amongst field collected *Ae*. *aegypti* and *Ae*. *albopictus* mosquitoes in Selangor and Kuala Lumpur in Peninsular Malaysia. In total, 31 samples were positive including dual infections in *Aedes* larvae. Co-infections of *Aedes* larvae with multiple serotypes of DENV have previously been reported in Brazil [34] and may have occurred as a result of transovarial transmission from the adult mosquito. Dual infections in adult *Ae*. *aegypti* and *Ae*. *albopictus* mosquitoes have also been found in Thailand [35]. The hyperendemicity of dengue in Malaysia presents an opportunity for co-infections, especially when taking into account the multiple feeding behaviour of *Ae*. *aegypti*. However, as the DENV co-infections were detected in larvae it is unclear if both serotypes would remain in the vector for transmission in adulthood. The single sample positive for DENV-2 that was detected in this study was part of a co-infection with sylvatic DENV-4. Due to the short length of the detected DENV-2 sequence, we are unable to ascertain the genotype of this sequence. The DENV-1 strain detected in this study belonged to genotype I which is related to strains circulating in neighbouring Singapore and Thailand. This particular strain was also the most prevalent serotype detected between 2005 and 2010 in Malaysia. More recently, genotype I was found to be the predominant strain among dengue patients in Selangor and Kuala Lumpur during the 2014–2016 epidemic period [36]. Non-severe dengue infections were more commonly observed in patients infected with this strain, unlike those infected with the Cosmopolitan genotype of DENV-2 that were more prone to severe clinical manifestations.

In Malaysia, the most common strain of DENV-3 has been from genotype III, which has circulated in the country for more than three decades and is one of the most widely distributed DENV genotypes globally [37]. All DENV-3-RNA-positive samples in the current study belonged to genotype V, a lineage that was identified by Wittke et al. [33] as a group of viruses that were previously classified as genotype I [38], but displayed amino acid changes that distinguished them from this genotype. Hence the DENV-3 viruses were separated into five main groups with the addition of the fifth genotype. Viruses belonging to genotype V include old Asian viruses and have not been associated with cases of DHF until recently, when a fatal case of DHF due to this genotype was identified in Brazil in 2003 [32]. The same genotype V virus was detected in patients in Colombia [39] and in field-captured *Ae*. *aegypti* mosquitoes in dengue-endemic areas in Thailand in 2011 [40]. Thus far, this genotype has not been reported in Malaysia. All DENV-3 isolates in this study were closely related to the early Asian isolates, however as the sequence available in this study was only from the CprM region of the genome a detailed assessment of the relationship between the different strains could not be carried out. Further study on the full sequence of this genotype will be necessary to determine the origins and pathogenicity of the virus.

DENV-4 has not been associated with major DENV epidemics in Malaysia since the 1960s [41] and appears to be circulating at a lower prevalence compared to the other three. In this study, sylvatic DENV-4 strains were detected in both *Ae*. *aegypti* and *Ae*. *albopictus* larvae collected from Selangor and Kuala Lumpur in Peninsular Malaysia. The large numbers of *Ae*. *albopictus* collected in urban areas in the study is concerning, as this vector has high potential to act as a ‘bridging vector’ and, in the absence of the primary vector *Ae*. *aegypti*, contribute to dengue epidemics amongst susceptible human populations [42]. Furthermore, the DENV-4 isolates in this study were determined by phylogenetic analysis to be a sylvatic strain that is very closely related to sylvatic DENV-4 sequences isolated from *Ae*. *niveus* (EF457906) and sentinel monkeys (JF262779 and JF262780) by Rudnick in Malaysia in the 1970s [9]. In West Africa, the forest-dwelling *Ae*. *furcifer* is known to circulate amongst rural villages and is capable of transmitting sylvatic DENV to resident human populations. In the study by Diallo and colleagues, this vector, along with other vectors such as *Ae*. *luteocephalus*, *Ae*. *vittatus* and *Ae*. *aegypti* were susceptible to infection with both sylvatic and urban strains of DENV-2, although the latter two vectors displayed a lower susceptibility to infection [43].

Evidence of the ability of sylvatic DENV to emerge from an enzootic cycle to infect humans, as well as their presence in urban vector populations in this study, raises concerns over the potential of these viruses to cause significant outbreaks in the future. There have been recent reports of human infections with highly divergent strains of DENV-1 [44] and DENV-2 [45,46] in travellers returning from Borneo to Australia. The ability of these suspected sylvatic strains to spill over and cause human disease raises significant public health concerns especially with increased travel and exposure of susceptible populations. The growing population of *Ae*. *albopictus* and its opportunistic feeding behaviour would make it the most likely vector for transmission of DENV to both primate and non-primate hosts which may act as virus reservoirs in urban settings [47–49]. However, thus far there is no experimental evidence of this route. The current study was also based on relatively short sequence lengths of less than 500nt and confined to two localities within the Klang Valley. Hence more extensive molecular epidemiology studies will be required to ascertain virus lineage and whether detected virus strains are also present in other parts of the country. It is unclear if the sylvatic DENV-4 lineage detected in *Ae*. *aegypti* and *Ae*. *albopictus* larva in this study will be present in adult mosquitoes for onwards transmission and subsequently cause similar disease in humans and warrants further work into the vector competence of vectors infected with this virus. Nevertheless, the possibility of such infections will need to be taken into account in future prevention and control programs in the country.

## Supporting information

S1 Table List of primers used for dengue virus nested PCR. (DOC)

S2 Table List of positive controls for DENV serotypes 1 to 4. (DOC)

S3 Table List of sequences of DENV serotypes 1 to 4 detected in *Aedes* mosquitoes from Selangor and Kuala Lumpur during 2016–2017. (DOC)
