# Supplementary material for: Sylvatic dengue virus type 4 in Aedes aegypti and Aedes albopictus mosquitoes in an urban setting in Peninsular Malaysia
Source: PLoS Negl Trop Dis. 2019 Nov 15;13(11):e0007889. doi: 10.1371/journal.pntd.0007889 (PMC6881067; doi:10.1371/journal.pntd.0007889)
Supplement: S1 Table — (DOC) [file pntd.0007889.s001.doc]

**S1 Table. List of primers used for dengue virus nested PCR.**

| **Sample** | **Primer** | **Primer sequence** |
| --- | --- | --- |
| DENV | D1 | 5’-TCA ATA TGC TGA AAC GCG CGA GAA ACC G |
|  | D2 | 5’-TTG CAC CAA CAG TCA ATG TCT TCA GGT TC |
|  | Dcon | 5’-AGT TGT TAG TCT ACG TGG ACC GAC A |
| DENV1 | TS1 | 5’-CGT CTC AGT GAT CCG GGG G |
| DENV2 | TS2 | 5’-CGC CAC AAG GGC CAT GAA CAG |
| DENV3 | TS3 | 5’-TAA CAT CAT CAT GAG ACA GAG C |
| DENV4 | TS4 | 5’-CTCTGTTGTCTTAAACAAGAGA |
