# Supplementary material for: Sylvatic dengue virus type 4 in Aedes aegypti and Aedes albopictus mosquitoes in an urban setting in Peninsular Malaysia
Source: PLoS Negl Trop Dis. 2019 Nov 15;13(11):e0007889. doi: 10.1371/journal.pntd.0007889 (PMC6881067; doi:10.1371/journal.pntd.0007889)
Supplement: S2 Table — (DOC) [file pntd.0007889.s002.doc]

**S2 Table. List of positive controls for** DENV serotypes 1 to 4.

| **DENV serotype** | **Positive control strain** | **Accession number** |
| --- | --- | --- |
| DENV-1 | US/Hawaii/1944 | EU848545 |
| DENV-2 | PNG/New Guinea C/1944 | AF038403 |
| DENV-3 | Philippines/H87/1956 | M93130 |
| DENV-4 | Philippines/H241/1956 | AY947539 |
