# Supplementary material for: Sylvatic dengue virus type 4 in Aedes aegypti and Aedes albopictus mosquitoes in an urban setting in Peninsular Malaysia
Source: PLoS Negl Trop Dis. 2019 Nov 15;13(11):e0007889. doi: 10.1371/journal.pntd.0007889 (PMC6881067; doi:10.1371/journal.pntd.0007889)
Supplement: S3 Table — (DOC) [file pntd.0007889.s003.doc]

**S3 Table. List of sequences of DENV serotypes 1 to 4 detected in *Aedes* mosquitoes from Selangor and Kuala Lumpur** during 2016-2017.

| **DENV Serotype** | **Area** | **Sample** | **Genbank Accession  Number** | **Host** | **Stage** | **DENV Genotype** | **Sequence length (nt)** |
| --- | --- | --- | --- | --- | --- | --- | --- |
| **1** | Selangor | USJT8G/2017 | MH377083 | *Ae. aegypti* | Adult | I | 429 |
| **2** | Kuala Lumpur | BJI29-2/2016 | MH377084 | *Ae. albopictus* | Larva | - | 70 |
| **3** | Selangor | USJA12Gb/2016 | MH377085 | *Ae. aegypti* | Larva | V | 240 |
|  |  | USJA14Gd/2016 | MH377086 | *Ae. aegypti* | Larva |  |  |
|  |  | USJA14e/2017 | MH377087 | *Ae. aegypti* | Larva |  |  |
|  |  | USJI14/2016 | MH377088 | *Ae. albopictus* | Larva |  |  |
|  |  | USJI15G/2016 | MH377089 | *Ae. albopictus* | Larva |  |  |
|  |  | USJB25G/2016 | MH377090 | *Ae. albopictus* | Larva |  |  |
|  | Kuala Lumpur | BJB26G/2016 | MH377091 | *Ae. albopictus* | Larva |  |  |
|  |  | BJD2-3/2016 | MH377092 | *Ae. albopictus* | Larva |  |  |
|  |  | BJF27/2016 | MH377093 | *Ae. albopictus* | Larva |  |  |
|  |  | BJG21/2016 | MH377094 | *Ae. albopictus* | Larva |  |  |
|  |  | BJG28/2016 | MH377095 | *Ae. albopictus* | Larva |  |  |
|  |  | BJG30/2016 | MH377096 | *Ae. albopictus* | Larva |  |  |
|  |  | BJH4G/2016 | MH377097 | *Ae. albopictus* | Larva |  |  |
| **4** | Selangor | USJA15Ge/2016 | MH377098 | *Ae. aegypti* | Larva | IV (Sylvatic) | 351 |
|  |  | USJA15Gg/2016 | MH377099 | *Ae. aegypti* | Larva |  |  |
|  |  | USJA15Gh/2016 | MH377100 | *Ae. aegypti* | Larva |  |  |
|  |  | USJA14a/2017 | MH377101 | *Ae. aegypti* | Larva |  |  |
|  |  | USJA14c/2017 | MH377102 | *Ae. aegypti* | Larva |  |  |
|  |  | USJA14Ga/2017 | MH377103 | *Ae. aegypti* | Larva |  |  |
|  |  | USJF8/2016 | MH377104 | *Ae. albopictus* | Larva |  |  |
|  |  | USJG24/2016 | MH377105 | *Ae. albopictus* | Larva |  |  |
|  | Kuala Lumpur | BJH8e/2016 | MH377106 | *Ae. aegypti* | Larva |  |  |
|  |  | BJH21a/2016 | MH377107 | *Ae. aegypti* | Larva |  |  |
|  |  | BJA27/2016 | MH377108 | *Ae. albopictus* | Larva |  |  |
|  |  | BJD2-4/2016 | MH377109 | *Ae. albopictus* | Larva |  |  |
|  |  | BJG10/2016 | MH377110 | *Ae. albopictus* | Larva |  |  |
|  |  | BJI29-4/2016 | MH377111 | *Ae. albopictus* | Larva |  |  |
|  |  | BJE19/2016 | MH377112 | *Ae. albopictus* | Larva |  |  |
|  |  | BJG15/2016 | MH377113 | *Ae. albopictus* | Larva |  |  |
|  |  | BJG19G/2016 | MH377114 | *Ae. albopictus* | Larva |  |  |
|  |  | BJA24/2016 | MH377115 | *Ae. albopictus* | Larva |  |  |
